# Vitamin A deficiency impairs neutrophil-mediated control of *Salmonella* via SLC11A1 in mice

**DOI:** 10.1038/s41564-024-01613-0

**Published:** 2024-02-19

**Authors:** Kristen L. Lokken-Toyli, Vladimir E. Diaz-Ochoa, Lizbeth Camacho, Annica R. Stull-Lane, Amber E. R. Van Hecke, Jason P. Mooney, Ariel D. Muñoz, Gregory T. Walker, Daniela Hampel, Xiaowen Jiang, Jasmine C. Labuda, Claire E. Depew, Stephen J. McSorley, Charles B. Stephensen, Renée M. Tsolis

**Affiliations:** 1 Department of Medical Microbiology and Immunology, University of California, Davis, Davis, CA USA; 2 Western Human Nutrition Research Center, US Department of Agriculture, Davis, CA USA; 3 Department of Nutrition, University of California, Davis, Davis, CA USA; 4 Department of Anatomy, Physiology and Cell Biology, School of Veterinary Medicine, University of California Davis, Davis, CA USA

**Keywords:** Bacterial infection, Infection

## Abstract

In sub-Saharan Africa, multidrug-resistant non-typhoidal *Salmonella* serovars are a common cause of fatal bloodstream infection. Malnutrition is a predisposing factor, but the underlying mechanisms are unknown. Here we show that vitamin A deficiency, one of the most prevalent micronutrient deficits afflicting African children, increases susceptibility to disseminated non-typhoidal *Salmonella* disease in mice and impairs terminal neutrophil maturation. Immature neutrophils had reduced expression of *Slc11a1*, a gene that encodes a metal ion transporter generally thought to restrict pathogen growth in macrophages. Adoptive transfer of SLC11A1-proficient neutrophils, but not SLC11A1-deficient neutrophils, reduced systemic *Salmonella* burden in *Slc11a1*^−/−^ mice or mice with vitamin A deficiency. Loss of terminal granulopoiesis regulator CCAAT/enhancer-binding protein ϵ (C/EBPϵ) also decreased neutrophil-mediated control of *Salmonella*, but not that mediated by peritoneal macrophages. Susceptibility to infection increased in *Cebpe*^−/−^ *Slc11a1*^+/+^ mice compared with wild-type controls, in an *Slc11a1*-expression-dependent manner. These data suggest that SLC11A1 deficiency impairs *Salmonella* control in part by blunting neutrophil-mediated defence.

## Abstract (web summary)

Vitamin A deficiency exacerbates invasive non-typhoidal *Salmonella* infection in mice, revealing a restrictive role for SLC11A1 in neutrophils.

## Main

Non-typhoidal *Salmonella* serovars, such as *Salmonella enterica* serovar (*S*.) Typhimurium, cause a self-limited gastroenteritis in individuals with an intact immune system. However, individuals with immunocompromising conditions such as HIV, chronic granulomatous disease, cancer chemotherapy and sickle disease are at elevated risk of developing life-threatening disseminated infections^1–11^, in which symptoms of diarrhoea are commonly absent^12–14^. One condition predisposing children in sub-Saharan Africa to bloodstream infection with non-typhoidal *Salmonella* serovars is malnutrition^6,7,9,15–18^, but how malnutrition impairs control of disseminated bacterial infections is poorly understood. As vitamin A deficiency (VAD) is one of the most prevalent micronutrient deficiencies in sub-Saharan Africa, with an estimated 48% of children under 5 years of age affected^19^, we investigated the role of VAD in the control of systemic *S*. Typhimurium infection in mice. We found that VAD impaired infection-induced development of neutrophils, known as granulopoiesis. This reduction in neutrophil granulopoiesis resulted in a population of immature neutrophils in infected tissues that were deficient in killing *Salmonella*. Expression of *Slc11a1* was significantly reduced in these immature neutrophils. SLC11A1, also known as NRAMP1, is a metal transporter known to restrict growth of intracellular pathogens via nutritional immunity in macrophages. In this Article, we show that mice deficient in the transcription factor CCAAT/enhancer-binding protein ϵ (C/EBPϵ), which express SLC11A1 in macrophages, but not neutrophils, had an impaired ability to control *S*. Typhimurium infection. Furthermore, adoptive transfer of SLC11A1-functional neutrophils to mice expressing a non-functional *Slc11a1*^*D169*^ allele improved the ability of these mice to control systemic *S*. Typhimurium infection. Together, these results show that SLC11A1 function in neutrophils contributes to controlling disseminated *S*. Typhimurium infection, thereby identifying a previously unappreciated role of SLC11A1 in neutrophil-mediated host defence.

## Results

### VAD diminishes resistance to invasive *Salmonella* infection

**Fig. 1:**
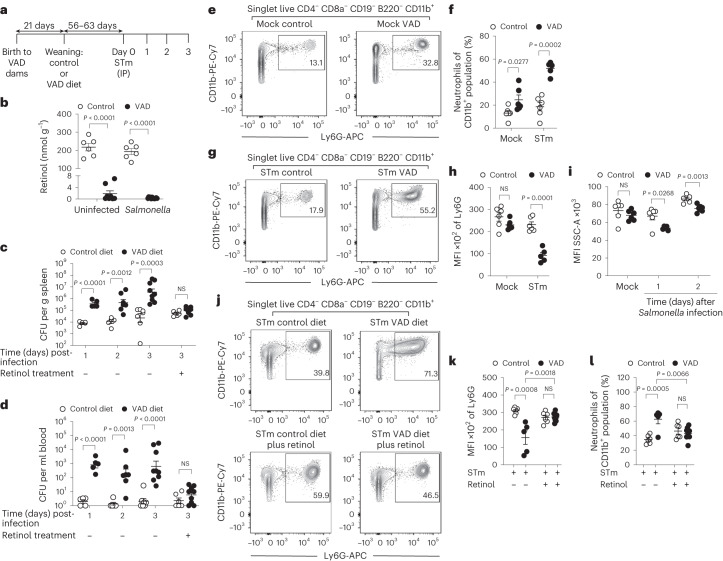
VAD increases systemic *S*. Typhimurium colonization and results in the accumulation of immature neutrophils in the spleen. **a**, Generation of VAD mice and experimental design. **b**, Hepatic retinol concentration in control mice (*n* = 6), VAD mice (*n* = 8), and control (*n* = 6) and VAD (*n* = 12) *Slc11a1*^*+/+*^ mice infected with *S*. Typhimurium, 3 days after infection. Statistical significance was determined on log-transformed values using one-way ANOVA with a post hoc Tukey test. **c**,**d**, *S*. Typhimurium colonization (CFU) of the spleen (**c**) and blood (**d**) at 1 day, 2 days and 3 days after infection of control (*n* = 5, *n* = 5, *n* = 7) and VAD (*n* = 5, *n* = 7, *n* = 9) *Slc11a1*^*+/+*^ mice, and control (*n* = 6) and VAD *Slc11a1*^*+/+*^ mice (*n* = 9) pretreated intragastrically with retinyl palmitate. Statistical significance was determined on log-transformed values using an unpaired, two-tailed Student’s *t*-test. **e**, Representative contour plots of splenic neutrophil frequency in male mock-infected control and VAD *Slc11a1*^*+/+*^ mice. Insets: gates used to quantify CD11b^+^ Ly6G^+^ cells. **f**, Frequency of splenic neutrophils in the CD11b^+^ population from control (*n* = 6, *n* = 6) and VAD (*n* = 6, *n* = 5) *Slc11a1*^*+/+*^ mice mock infected and infected with *S*. Typhimurium (1 day). Statistical significance was determined on arc-sin-transformed values using an unpaired, two-tailed Student’s *t-*test. **g**, Representative contour plots of splenic neutrophil frequency in male control and VAD *Slc11a1*^*+/+*^ mice infected with *S*. Typhimurium (1 day). **h**, Median fluorescence intensity (MFI) of surface Ly6G on splenic neutrophils of control (*n* = 6, *n* = 6) and VAD (*n* = 6, *n* = 5) *Slc11a1*^*+/+*^ mice mock infected and infected with *S*. Typhimurium (1 day). **i**, Side scatter area of splenic neutrophils from control (1 day, *n* = 6; 2 days, *n* = 5) and VAD (1 day, *n* = 5; 2 days, *n* = 7) *Slc11a1*^*+/+*^ mice mock infected (*n* = 6) and infected (*n* = 6) with *S*. Typhimurium. In **h** and **i**, statistical significance was determined on log-transformed values using an unpaired, two-tailed Student’s *t-*test. **j**, Representative contour plots of splenic neutrophil frequency of male control and VAD *Slc11a1*^*+/+*^ mice infected with *S*. Typhimurium and pretreated intragastrically with PBS or retinyl palmitate. **k**,**l**, MFI of surface Ly6G (**k**) and frequency (**l**) of splenic neutrophils from control and VAD *Slc11a1*^*+/+*^ mice infected with *S*. Typhimurium and pretreated intragastrically with PBS (*n* = 6, *n* = 5) or retinyl palmitate (*n* = 6, *n* = 9). Statistical significance was determined on log- or arc-sin-transformed values using one-way ANOVA with a post hoc Tukey test. All data represent individual mice with mean ± s.e.m. and are collected from one experiment. NS, not significant; STm, *S*. Typhiumurium; SSC-A, side scatter area. Source data

**Extended Data Fig. 1:**
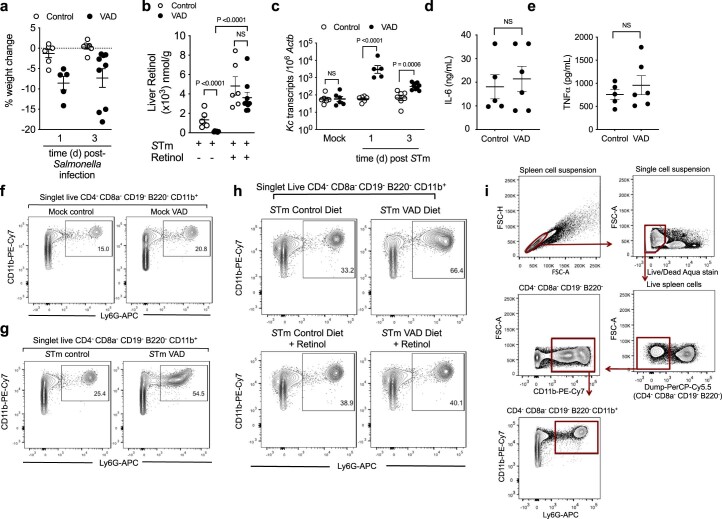
Recruitment of immature neutrophils to the spleen. **a**, Average percent weight change at 1- and 3-days post- *S*. Typhimurium infection of control (n = 6; n = 7) and VAD (n = 5; n = 9) *Slc11a1*^*+/+*^ mice. Data collected from one experiment. **b**, Hepatic retinol concentration in control and VAD *Slc11a1*^*+/+*^ mice treated intragastrically (IG) with PBS (n = 6; n = 6) or retinyl palmitate (n = 6; n = 9). Statistical significance was determined on log-transformed values using a one-way analysis of variance (ANOVA) with a post-hoc Tukey test. NS, not significant. Data are collected from one experiment. **c**, Expression of *Kc* in splenic tissue of mock-infected (n = 6 each) and *S*. Typhimurium-infected control (1 d n = 6; 3 d n = 7) and VAD (1 d n = 5; 3 d n = 9) *Slc11a1*^*+/+*^ mice. Statistical significance was determined on log-transformed values using an unpaired, two-tailed Student’s t test. NS, not significant. Data are collected from one experiment. **d-e**, Production of IL-6 (**d**) or TNFα (**e**) by control (n = 5) and VAD (n = 6) *Slc11a1*^*+/+*^ mice 2 h after IP injection with 20 µg LPS. Statistical significance was determined on log-transformed values using an unpaired, two-tailed Student’s t test. NS, not significant. Data are collected from one experiment. **f-g**, Representative contour plots of splenic neutrophil frequency (singlet live CD4-CD8a-CD19-B220-CD11b + Ly6G + ) from mock-infected female *Slc11a1*^*+/+*^ mice fed control or VAD diets (**f**) or *Slc11a1*^*+/+*^ mice infected for 1d with *S*. Typhimurium **(g)**. **h**, Representative contour plots of splenic neutrophil frequency (singlet live CD4-CD8a-CD19-B220-CD11b + Ly6G+) from female *S*. Typhimurium-infected (3d) control and VAD *Slc11a1*^*+/+*^ mice pretreated with either PBS or retinyl palmitate (600 IU delivered IG) at 7d and 3d before *S*. Typhimurium infection. **i**, Gating strategy for analysis of bone marrow and spleen single cell suspensions. After doublet elimination, live cells were gated and CD4-CD8a-CD19-B220- Ter119-, for bone marrow analysis, and CD4-CD8a-CD19-B220- for spleen analysis, were analyzed for CD11b and Ly6G expression. Neutrophils were defined as CD11b + Ly6G + . All data represent individual mice with the mean ± SEM. Source data

We used a mouse model to determine whether VAD impairs control of systemic infection with *S*. Typhimurium strain JK1128 (ref. ^20^; Fig. 1a), a strain belonging to the ST313 lineage currently circulating in sub-Saharan Africa^21^. VAD was induced in *Slc11a1*^*+/+*^ C57BL/6J mice, which are genetically resistant to *S*. Typhimurium infection^22^. Mice with VAD (hereafter VAD mice) had depleted hepatic retinol stores, compared with conventionally raised control mice (Fig. 1b). *S*. Typhimurium infection resulted in greater weight loss (Extended Data Fig. 1a) and more than 100-fold higher bacterial burden in the spleen (Fig. 1c) and blood (Fig. 1d) of VAD mice compared with control mice. The increased susceptibility of VAD mice was evident by the first day of *S*. Typhimurium infection (Fig. 1c,d), pointing to an innate immune defect as a possible driver of increased systemic pathogen burden. Treatment with the vitamin A supplement retinyl palmitate before *S*. Typhimurium infection restored control of systemic pathogen growth. While vitamin A supplementation had no effect on bacterial numbers recovered from control mice, the treatment restored liver retinol levels (Extended Data Fig. 1b) and significantly (*P* < 0.05) improved the ability of VAD mice to control bacterial burden in the spleen (Fig. 1c) and blood (Fig. 1d). Collectively, these data indicated that VAD led to a prominent, but reversible, impairment of the host’s innate ability to control disseminated *S*. Typhimurium infection.

We have recently shown that malaria increases the risk of *S*. Typhimurium bacteraemia by blunting expression of neutrophil chemoattractants and reducing neutrophil recruitment during *S*. Typhimurium infection^23^. By contrast, in the spleen of VAD mice, *S*. Typhimurium infection triggered higher transcript levels of *Kc* (encoding KC, or keratinocyte-derived cytokine) compared with control mice (Extended Data Fig. 1c), which suggested that VAD did not impair control of disseminated *S*. Typhimurium infection by blunting the expression of the neutrophil chemoattractant KC. Furthermore, VAD mice also produced interleukin-6 (IL-6) and tumour necrosis factor-α (TNF-α) in an equivalent manner to mice receiving a control diet in response to lipopolysaccharide (LPS) injection, further showing their ability to respond to bacterial ligands (Extended Data Fig. 1d,e).

In the absence of infection, the splenic phagocyte population (CD4^−^ CD8a^−^ CD19^−^ B220^−^ CD11b^+^ splenocytes) of VAD mice contained a significantly (*P* < 0.05) larger proportion of neutrophils (CD4^−^ CD8a^−^ CD19^−^ B220^−^ CD11b^+^ Ly6G^+^ splenocytes) compared with control mice (Fig. 1e,f and Extended Data Fig. 1f). Furthermore, during *S*. Typhimurium infection, the abundance of infiltrating neutrophils was significantly (*P* < 0.05) increased in VAD mice compared with controls (Fig. 1f,g and Extended Data Fig. 1e). Collectively, these data suggested that unlike malaria, VAD did not increase susceptibility to *S*. Typhimurium infection by impairing neutrophil recruitment.

Interestingly, while surface expression of lymphocyte antigen 6 complex locus G6D (Ly6G) was not affected by vitamin A status in uninfected mice, neutrophils in the spleen of VAD mice infected with *S*. Typhimurium exhibited significantly lower median Ly6G fluorescence intensity and reduced side scatter (a measure of granularity) than neutrophils in the spleen of control mice infected with *S*. Typhimurium (Fig. 1h,i). Retinyl palmitate treatment before *S*. Typhimurium infection restored median Ly6G fluorescence intensity in VAD mice (Fig. 1j,k) and reduced the abundance of neutrophils in VAD mice to levels in control mice (Fig. 1l). As both median Ly6G fluorescence intensity and granule development increase as neutrophils mature^24^, these results raised the possibility that VAD might weaken immunity by compromising neutrophil maturation during *S*. Typhimurium infection.

### Impairment of infection-induced granulopoiesis in VAD mice

**Fig. 2:**
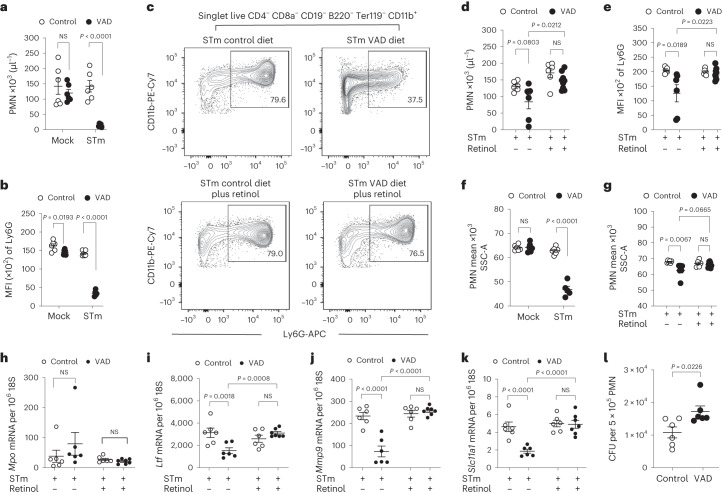
VAD impairs granulopoiesis induced by systemic *S*. Typhimurium infection. **a**,**b**, Number of bone marrow polymorphonuclear neutrophils (PMN; singlet live CD4^−^ CD8a^−^ CD19^−^ B220^−^ Ter119^−^ CD11b^+^ Ly6G^+^) (**a**) and MFI of surface Ly6G (**b**) from control (*n* = 6, *n* = 6) and VAD (*n* = 6, *n* = 5) *Slc11a1*^*+/+*^ mice mock infected and infected with *S*. Typhimurium (1 day). Statistical significance was determined on log-transformed values using an unpaired, two-tailed Student’s *t*-test. Data are collected from one experiment. **c**, Representative contour plots of bone marrow neutrophil frequency from male control and VAD *Slc11a1*^*+/+*^ mice infected with *S*. Typhimurium and pretreated intragastrically with PBS or retinyl palmitate. **d**,**e**, Number of bone marrow neutrophils (**d**) and MFI of surface Ly6G (**e**) from control and VAD *Slc11a1*^*+/+*^ mice infected with *S*. Typhimurium and pretreated intragastrically with PBS (*n* = 6, *n* = 6) or retinyl palmitate (*n* = 6, *n* = 9). Statistical significance was determined on log-transformed values using a one-way ANOVA with a post hoc Tukey test. Data are collected from one experiment. **f**, Side scatter area of bone marrow neutrophils (singlet live CD4^−^ CD8a^−^ CD19^−^ B220^−^ Ter119^−^ CD11b^+^ Ly6G^+^) from control (*n* = 6, *n* = 6) and VAD (*n* = 6, *n* = 5) *Slc11a1*^*+/+*^ mice mock infected and infected with *S*. Typhimurium (1 day). Statistical significance was determined on log-transformed values using an unpaired, two-tailed Student’s *t-*test. Data are collected from one experiment. **g**, Side scatter area of bone marrow neutrophils from control and VAD *Slc11a1*^*+/+*^ mice infected with *S*. Typhimurium and pretreated intragastrically with PBS (*n* = 6, *n* = 6) or retinyl palmitate (*n* = 6, *n* = 9). Statistical significance was determined on log-transformed values using a one-way ANOVA with a post hoc Tukey test. Data are collected from one experiment. **h**–**k**, Expression of myeloperoxidase (*Mpo*) (**h**), lactoferrin (*Ltf*) (**i**), matrix metallopeptidase 9 (*Mmp9*) (**j**) and *Slc11a1* (**k**) in bone marrow neutrophils isolated from control and VAD *Slc11a1*^*+/+*^ mice infected with *S*. Typhimurium and pretreated intragastrically with PBS (*n* = 6, *n* = 6) or retinyl palmitate (*n* = 6, *n* = 7). Transcript numbers were normalized to 18S ribosomal RNA (18S). Statistical significance was determined on log-transformed values using a one-way ANOVA with a post hoc Tukey test. Data are collected from two independent experiments. **l**, Intracellular *S*. Typhimurium recovered 2 h after ex vivo infection of bone marrow PMN from control (*n* = 6) and VAD (*n* = 6) *Slc11a1*^*+/+*^ mice. Statistical significance was determined on log-transformed values using an unpaired, two-tailed Student’s *t-*test. Data were collected from one experiment. All data represent individual mice with mean ± s.e.m. Source data

**Extended Data Fig. 2:**
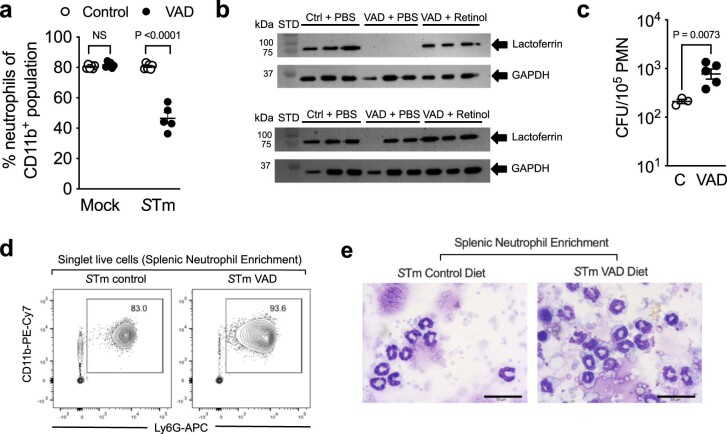
VAD impairs emergency granulopoiesis in response to *S*. Typhimurium infection. **a**, Frequency of bone marrow neutrophils (singlet live CD4-CD8a-CD19-B220-Ter119-CD11b + Ly6G+) from control and VAD *Slc11a1*^*+/+*^ mice at 1d after *S*. Typhimurium infection (n = 6; n = 5) or mock treatment (n = 6; n = 6). Statistical significance was determined on arc-sin-transformed values using an unpaired, two-tailed Student’s *t* test. NS, not significant. Data are collected from one experiment. Lactoferrin levels (**b**) in bone marrow neutrophils isolated from PBS treated control (n = 6) and VAD (n = 6) *Slc11a1*^*+/+*^ mice, and retinyl palmitate treated VAD (n = 6) *Slc11a1*^*+/+*^ mice. Data are collected from two independent experiments. STD, protein standard. **c**, CFU of *S*. Typhimurium associated with a neutrophil enriched splenic cell suspension from control (n = 3) and VAD (n = 5) *Slc11a1*^*+/+*^ mice 3d post-infection. Statistical significance was determined on log-transformed values using an unpaired, two-tailed Student’s *t* test. Data are collected from one experiment. **d**, Representative contour plot of neutrophils enriched from the spleen of *S*. Typhimurium infected control and VAD *Slc11a1*^*+/+*^ mice showing greater than 83% neutrophil purity (singlet live CD11b + Ly6G+). **e**, Representative images of Giemsa-stained Ly6G+ neutrophils enriched from the spleen of S. Typhimurium infected control and VAD *Slc11a1*^*+/+*^ mice. Scale bar = 50μm Each image represents one *S*. Typhimurium-infected control and VAD animal from one experiment. All data represent individual mice with the mean ± SEM. Source data

During bacterial infection, the host responds by rapidly mobilizing and increasing de novo production of neutrophils in the bone marrow to levels beyond steady-state conditions, a process termed emergency granulopoiesis^25^. We thus wanted to determine whether VAD would impair infection-driven granulopoiesis in the bone marrow. In the absence of infection, the bone marrow of VAD and control mice contained similar numbers of neutrophils (Fig. 2a and Extended Data Fig. 2a) of comparable maturity, as indicated by similar median Ly6G fluorescence intensities (Fig. 2b), suggesting that steady-state granulopoiesis was not impaired during VAD. In contrast, after *S*. Typhimurium infection, the bone marrow of VAD mice contained lower numbers of neutrophils (Fig. 2a–d and Extended Data Fig. 2a) than that of control mice. Furthermore, bone marrow neutrophils from VAD mice infected with *S*. Typhimurium exhibited significantly (*P* < 0.05) lower median Ly6G fluorescence intensity (Fig. 2b,c,e) and granularity (Fig. 2f,g) than those from controls. Supplementation with retinol (as retinyl palmitate) restored numbers (Fig. 2c,d) as well as median Ly6G fluorescence intensity (Fig. 2e) and granularity (Fig. 2g) of neutrophils in the bone marrow of VAD mice infected with *S*. Typhimurium, suggesting that VAD impaired infection-driven granulopoiesis. Neutrophils in the bone marrow of VAD mice infected with *S*. Typhimurium showed significantly reduced granularity (determined by side scatter, Fig. 2f), indicative of impaired granule formation, which was restored to control levels by retinyl palmitate supplementation (Fig. 2g). Neutrophil development is characterized by a sequential formation of distinct granule subsets, which proceeds through the formation of azurophil granules (during the promyelocyte stage), specific granules (during the myelocyte and metamyelocyte stages) and gelatinase granules (during band and segmented stages)^26^. While expression of *Mpo*, encoding the azurophil granule protein myeloperoxidase, remained unchanged (Fig. 2h), transcript levels of *Ltf*, encoding the specific granule protein lactoferrin (Fig. 2i), and *Mmp9*, encoding the gelatinase granule protein matrix metalloproteinase 9 (Fig. 2j), were significantly (*P* < 0.05) diminished in bone marrow neutrophils of VAD mice infected with *S*. Typhimurium compared with those of controls. Expression of both *Ltf* and *Mmp9* could be restored to control levels by retinyl palmitate supplementation (Fig. 2i,j). Furthermore, bone marrow neutrophil lactoferrin levels were undetectable in four of six VAD mice infected with *S*. Typhimurium and were subsequently restored with previous retinyl palmitate supplementation (Extended Data Fig. 2b). Together, these results suggested that VAD inhibited early control of systemic *S*. Typhimurium infection by reducing terminal neutrophil differentiation. Interestingly, expression of *Slc11a1*, encoding an additional component of neutrophil gelatinase granules^27^, was significantly reduced in bone marrow neutrophils of VAD mice infected with *S*. Typhimurium compared with controls (Fig. 2k), whereas supplementation of VAD mice with retinyl palmitate before *S*. Typhimurium infection restored expression of *Slc11a1* to control levels (Fig. 2k). *Slc11a1* encodes a phagosomal transporter of Fe^2+^ and Mn^2+^, which has been proposed to reduce the availability of these metals, as well as that of Mg^2+^, to *Salmonella*^28,29^. Production of a non-functional SLC11A1^G169D^ variant in mouse macrophages increases susceptibility to intracellular pathogens, such as *S*. Typhimurium^30,31^, presumably via withholding of divalent metals from the pathogen-containing phagosome^32,33^. However, while mature human neutrophils are known to produce high levels of SLC11A1 (ref. ^34^), its role in neutrophil function is not known. We thus wanted to investigate whether VAD impairs a hitherto unknown SLC11A1-dependent antimicrobial activity of neutrophils.

### Effect of VAD on *S*. Typhimurium infection requires SLC11A1

**Fig. 3:**
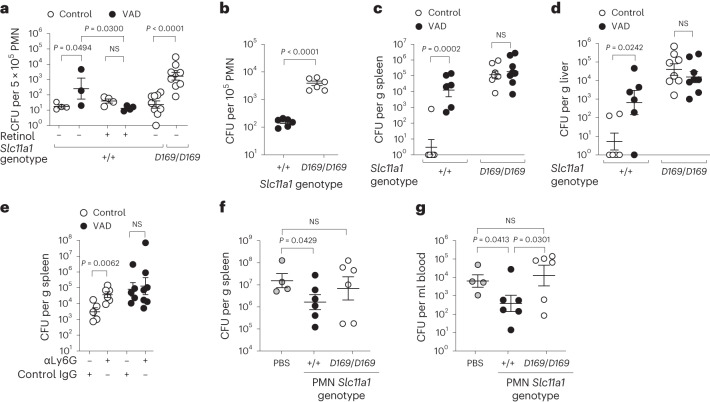
Functional SLC11A1 contributes to neutrophil-mediated control of *S*. Typhimurium infection. **a**, CFU of *S*. Typhimurium associated with a neutrophil-enriched splenic cell suspension 3 days after infection from control and VAD *Slc11a1*^*+/+*^ mice pretreated intragastrically with PBS (*n* = 4, *n* = 3) or retinyl palmitate (*n* = 4, *n* = 4). Statistical significance was determined on log-transformed values using a one-way ANOVA with a post hoc Sidak test. Data were collected from one experiment. CFU of *S*. Typhimurium associated with a neutrophil-enriched splenic cell suspension 3 days after infection from control *Slc11a1*^*+/+*^ (*n* = 9) and *Slc11a1*^*D169/D169*^ (*n* = 9) mice. Statistical significance was determined on log-transformed values using an unpaired, two-tailed Student’s *t-*test. Data were collected from one experiment. **b**, Ex vivo killing of *S*. Typhimurium by bone marrow PMN from *Slc11a1*^*+/+*^ (*n* = 6) and *Slc11a1*^*D169/D169*^ (*n* = 6) mice. Statistical significance was determined on log-transformed values using an unpaired, two-tailed Student’s *t-*test. Data were collected from three independent experiments. **c**,**d**, *S*. Typhimurium colonization of the spleen (**c**) and liver (**d**) 3 days after oral administration (IG) of *S*. Typhimurium to control and VAD *Slc11a1*^*+/+*^ (*n* = 6, *n* = 6) and *Slc11a1*^*D169/D169*^ (*n* = 8, *n* = 8) mice. Statistical significance was determined on log-transformed values using an unpaired, two-tailed Student’s *t-*test. Data were collected from one experiment. **e**, *S*. Typhimurium colonization of the spleen 2 days after IP infection. Control and VAD male *Slc11a1*^*+/+*^ mice were treated intraperitoneally with a neutrophil-depleting antibody (αLy6G, *n* = 6, *n* = 7) or isotype control (*n* = 5, *n* = 5) 1 day before and 1 day after infection. Statistical significance was determined on log-transformed values using an unpaired, two-tailed Student’s *t-*test. Data were collected from two independent experiments. **f**,**g**, Colonization of the spleen (**f**) and blood (**g**) 3 days after IP infection of *Slc11a1*^*+/+*^ VAD male mice that received 3–4 × 10^6^ bone marrow PMN from either *Slc11a1*^*D169/D169*^ (*n* = 6) or *Slc11a1*^*+/+*^ (*n* = 6) mice, or PBS (*n* = 4) administered intraperitoneally 1 day after *S*. Typhimurium infection. Statistical significance was determined on log-transformed values using an unpaired, one-tailed Student’s *t-*test. Data were collected from two independent experiments. All data represent individual mice with mean ± s.e.m. Source data

Neutrophils from VAD mice infected ex vivo (Fig. 2l) or enriched from VAD mice infected with *S*. Typhimurium (Fig. 3a and Extended Data Fig. 2c) contained significantly more bacteria compared with neutrophils from infected mice on a control diet, and pretreatment of mice with retinyl palmitate before *S*. Typhimurium infection significantly reduced intracellular bacteria within neutrophils of VAD mice (Fig. 3a). To exclude the possibility that contaminating macrophages in the neutrophil-enriched cell suspension contributed to the differences in bacterial numbers, we characterized the cell suspension using flow cytometry and cytospins. Flow cytometry analysis of the neutrophil-enriched population showed >83% of the singlet live splenic cells to be neutrophils (CD11b^+^ Ly6G^+^) in both control and VAD mice infected with *S*. Typhimurium (Extended Data Fig. 2d). Cytospin analysis showed the majority of cells with nuclei of band or segmented shape, a defining characteristic of neutrophils (Extended Data Fig. 2e). C57BL/6J mice carry an allele (*Slc11a1*^*D169*^) that renders SLC11A1 nonfunctional, while the mice used for this study carry a functional *Slc11a1* allele (*Slc11a1*^*+/+*^)^22^. We therefore used these two strains of C57BL/6J mice to determine whether neutrophil expression of *Slc11a1* impacted control of *S*. Typhimurium. Neutrophils enriched from *Slc11a1*^*D169/D169*^ mice infected with *S*. Typhimurium contained significantly higher bacterial numbers compared with those from *Slc11a1*^*+/+*^ controls, indicating that *Slc11a1* expression in neutrophils enhanced resistance to *S*. Typhimurium (Fig. 3a). Neutrophils enriched from the bone marrow of uninfected *Slc11a1*^*D169/D169*^ mice and infected with *S*. Typhimurium ex vivo contained 100-fold greater numbers of *S*. Typhimurium compared with those from *Slc11a1*^*+/+*^ controls (Fig. 3b). Together, these results show that expression of a non-functional *Slc11a1* allele impairs the ability of neutrophils to control systemic *S*. Typhimurium infection.

The hypothesis that reduction of SLC11A1 synthesis in neutrophils is one of the mechanisms by which VAD impairs immunity against *S*. Typhimurium would predict that VAD does not alter control of *S*. Typhimurium infection in *Slc11a1*^*D169/D169*^ mice. To test this, we infected VAD mice and *Slc11a1*^*D169/D169*^ mice replete with vitamin A via the oral route and assessed systemic colonization 3 days after *S*. Typhimurium infection. VAD increased the bacterial burden in the spleen (Fig. 3c) and liver (Fig. 3d) of mice with an intact *Slc11a1* allele, but not in *Slc11a1*^*D169/D169*^ mice. These results suggested that one mechanism by which VAD promoted invasive *S*. Typhimurium infection is a reduction in SLC11A1-mediated defences.

**Extended Data Fig. 3:**
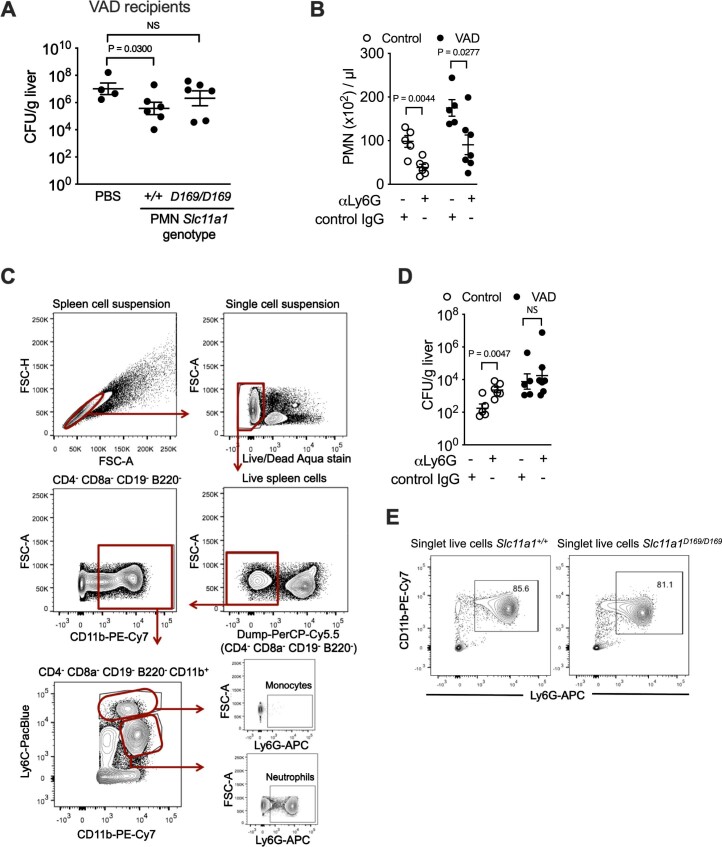
SLC11A1 contributes to neutrophil-mediated control of infection. **a**, Colonization of the liver at 3 d post IG infection of male VAD *Slc11a1*^*+/+*^ mice that received 3-4 × 10^6^ bone marrow neutrophils from either *Slc11a1*^*D169/D169*^ (n = 6) or *Slc11a1*^*+/+*^ (n = 6) mice or PBS (n = 4) administered IP 1 d post-*S*. Typhimurium infection. Statistical significance was determined on log-transformed values using an unpaired, one-tailed Student’s *t* test. NS, not significant. Data are collected from two independent experiments. **b**, Number of splenic neutrophils (CD11b+Ly6Cmid) 2d post-*S*. Typhimurium infection from male control and VAD *Slc11a1*^*+/+*^ mice treated with a neutrophil tissue depleting antibody (anti-Ly6G; 1A8) (n = 6; n = 7) or IgG control antibody (n = 5; n = 5) administered one-day prior and after *S*. Typhimurium infection. Statistical significance was determined on log-transformed values using an unpaired, two-tailed Student’s *t* test. Data are collected from two independent experiments. **c**, Gating strategy for analysis of spleen single cell suspensions after neutrophil depletion. After doublet elimination, live cells were gated and CD4-CD8a-CD19-B220- cells were analyzed for CD11b and Ly6C expression. Neutrophils were defined as CD11b+Ly6Cmid and monocytes were defined as CD11b+Ly6Chi. **d**, *S*. Typhimurium colonization of the liver 2 d post infection from control and VAD *Slc11a1*^*+/+*^ mice treated with a neutrophil depleting antibody (n = 6; n = 7) or isotype control (n = 5; n = 5) one day prior and after infection. Statistical significance was determined on log-transformed values using an unpaired, two-tailed Student’s *t* test. NS, not significant. Data are collected from two independent experiments. **e**, Representative contour plots from flow cytometry analysis of neutrophil adoptive transfer showing 85% neutrophil purity (singlet live CD11b + Ly6G + ) from bone marrow isolation of healthy *Slc11a1*^*+/+*^ and *Slc11a1*^*D169/D169*^ donor mice. All data represent individual mice with the mean ± SEM. Source data

To determine directly whether reduced neutrophil function compromises host defence against *S*. Typhimurium infection during VAD, we restricted neutrophil extravasation by treatment with the Ly6G-specific monoclonal antibody 1A8 (ref. ^35^). Treatment with 1A8 significantly reduced neutrophil numbers in the spleen of both VAD and control mice compared with isotype controls (Extended Data Fig. 3b,c). Neutrophil depletion significantly increased recovery of *S*. Typhimurium from the spleen and liver of control mice (Fig. 3e and Extended Data Fig. 3d), suggesting that neutrophils were important for controlling *S*. Typhimurium infection in animals replete with vitamin A. By contrast, neutrophil depletion did not alter *S*. Typhimurium numbers recovered from VAD mice (Fig. 3e and Extended Data Fig. 3d), indicating that neutrophils no longer contributed to protection during VAD. Strikingly, adoptive transfer of bone-marrow-derived neutrophils (Extended Data Fig. 3f) isolated from healthy *Slc11a1*^*+/+*^ mice to VAD mice infected with *S*. Typhimurium significantly reduced bacterial burden in the spleen (Fig. 3f) and blood (Fig. 3g) compared with VAD controls. In contrast, adoptive transfer of bone marrow neutrophils from healthy *Slc11a1*^*D169/D169*^ mice into infected VAD mice failed to restore control of *S*. Typhimurium growth at systemic sites (Fig. 3f,g and Extended Data Fig. 3b). Taken together, these results suggested that one of the mechanisms by which lack of vitamin A compromises control of disseminated *S*. Typhimurium infection is the generation of a population of SLC11A1-deficient neutrophils that are unable to clear bacteria at systemic sites.

### SLC11A1 in neutrophils controls *Salmonella* infection

**Fig. 4:**
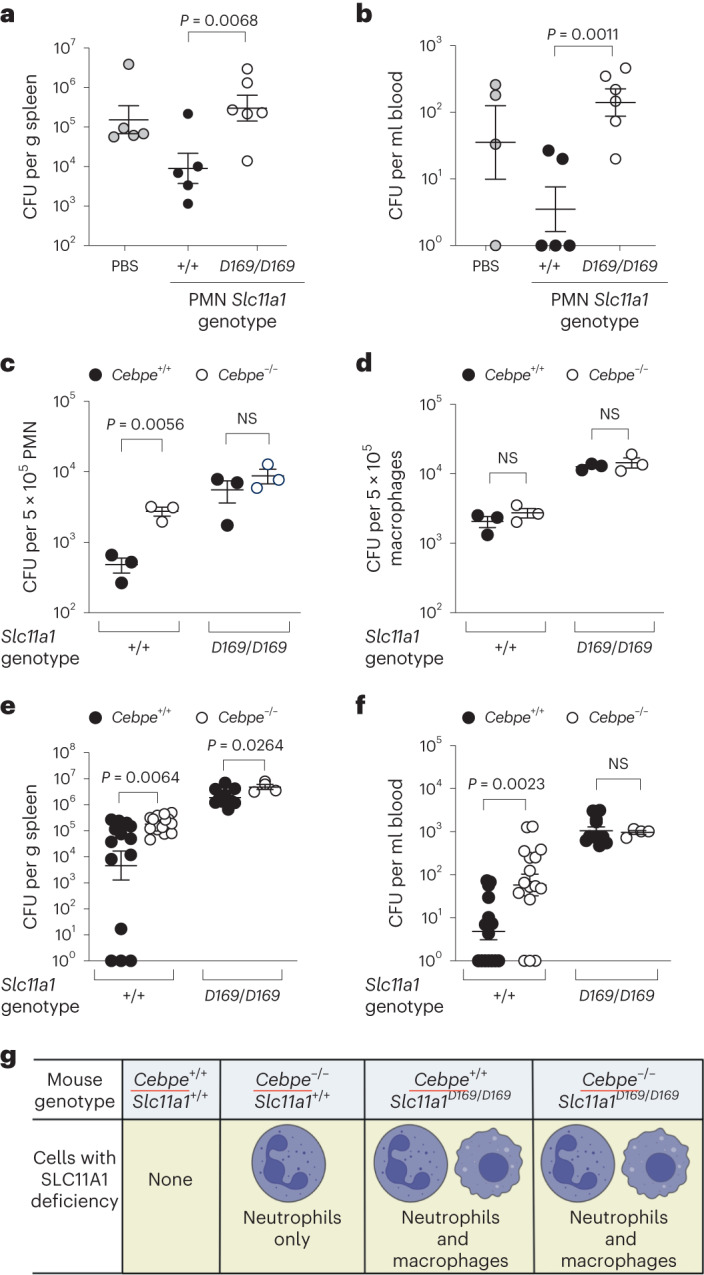
Mice conditionally deficient in SLC11A1 expression in neutrophils have impaired control of systemic *Salmonella* infection. **a**,**b**, Colonization of the spleen (**a**) and blood (**b**) 3 days after IG infection of *Slc11a1*^*D169/D169*^ male mice that received 5 × 10^6^ bone marrow PMN from either *Slc11a1*^*D169/D169*^ (*n* = 6) or *Slc11a1*^*+/+*^ (*n* = 5) mice or PBS (*n* = 5 or 4) administered intraperitoneally 1 day after *S*. Typhimurium infection. Statistical significance was determined on log-transformed values using an unpaired, one-tailed Student’s *t-*test. Data were collected from two independent experiments. **c**,**d**, Ex vivo killing of *S*. Typhimurium by bone marrow neutrophils (**c**) or elicited peritoneal macrophages (**d**) isolated from *Slc11a1*^*+/+*^ *Cebpe*^*+/+*^ (*n* = 3), *Slc11a1*^*+/+*^ *Cebpe*^*−/−*^ (neutrophil-specific *Slc11a1* deficiency; *n* = 3) and global *Slc11a1*-deficient, *Slc11a1*^*D169/D169*^ *Cebpe*^*+/+*^ (*n* = 3) and *Slc11a1*^*D169/D169*^ *Cebpe*^*−*^^*/*^^*−*^ (*n* = 9, *n* = 3) mice. Statistical significance was determined on log-transformed values using an unpaired, two-tailed Student’s *t-*test. Data were collected from three independent experiments, each performed with triplicate samples. **e**,**f**, *S*. Typhimurium colonization of the spleen (**e**) and blood (**f**) 3 days after IP infection of *Slc11a1*^*+/+*^ *Cebpe*^*+/+*^ (*n* = 15), *Slc11a1*^*+/+*^ *Cebpe*^*−*^^*/*^^*−*^(neutrophil-specific *Slc11a1* deficiency; *n* = 16) and global *Slc11a1*-deficient, *Slc11a1*^*D169/D169*^ *Cebpe*^*+/+*^ (*n* = 11) and *Slc11a1*^*D169/D169*^ *Cebpe*^*−*^^*/*^^*−*^ (*n* = 4) mice. Statistical significance was determined on log-transformed values using an unpaired, two-tailed Student’s *t-*test. Data were collected from one experiment. All data represent individual mice with mean ± s.e.m. **g**, Genotypes of mice used for panels **c**–**f** and cell types with SLC11A1 deficiency. Figure **g** created with BioRender.com. Source data

**Extended Data Fig. 4:**
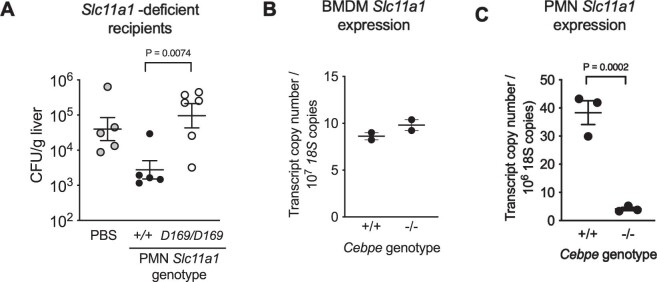
Expression and function of SLC11A1 in phagocytes. **a**, Colonization of the liver at 3 d post IG infection of *Slc11a1*^*D169/D169*^ mice that received 5 × 10^6^ bone marrow neutrophils from either *Slc11a1*^*D169/D169*^ (n = 6) or *Slc11a1*^*+/+*^ (n = 5) mice or PBS (n = 5) administered IP 1d post-*S*. Typhimurium infection. Statistical significance was determined on log-transformed values using an unpaired, one-tailed Student’s *t* test. Data are collected from two independent experiments. **b**, **c**, Expression of *Slc11a1* in macrophages (**b**) or neutrophils (**c**) derived from bone marrow of C/EBP epsilon-deficient mice (n = 2; n = 3) or littermate controls (n = 2; n = 3), as determined by qRT-PCR. Statistical significance was determined on log-transformed values using an unpaired, two-tailed Student’s *t* test. All data represent individual mice with the mean ± SEM. Source data

To determine whether SLC11A1 function in neutrophils contributes to control of systemic *Salmonella* infection, we performed adoptive transfer of bone marrow neutrophils isolated from healthy *Slc11a1*^*+/+*^ or *Slc11a1*^*D169/D169*^ mice to *Slc11a1*^*D169/D169*^ mice infected with *S*. Typhimurium. For these experiments, as neutrophils were transferred intraperitoneally, we changed the route of infection to the intragastric (IG) route to maximize the possibility that the transferred neutrophils would have to migrate to sites of infection to exert control of *S*. Typhimurium. Mice receiving *Slc11a1*^*+/+*^ neutrophils had significantly reduced bacterial burden in the spleen (Fig. 4a), blood (Fig. 4b) and liver (Extended Data Fig. 4a) compared with mice receiving *Slc11a1*^*D169/D169*^ neutrophils.

A possible contributor to reduced *Slc11a1* expression in neutrophils of VAD mice is that C/EBPϵ, which directs the terminal stages of granulopoiesis, is induced by retinoic acid^36^. To further study the role of neutrophil *Slc11a1* in the control of *Salmonella* infection by neutrophils, we crossed a defective allele of *Cebpe*^37^, encoding C/EBPϵ, onto our *Slc11a1*^*+/+*^ background mice. As *Slc11a1* is expressed only in mature neutrophils^38^, C/EBPϵ-deficient neutrophils lack *Slc11a1* expression (Extended Data Fig. 4b,c). We therefore hypothesized that mice resulting from this cross have SLC11A1 functional macrophages, but SLC11A1-deficient neutrophils. In an *Slc11a1*^*+/+*^ background, deficiency of *Cebpe* reduced the ability of neutrophils to kill *S*. Typhimurium ex vivo (Fig. 4c). By contrast, on an *Slc11a1*-deficient background (*Slc11a1*^*D169/D169*^), no effect of *Cebpe* deficiency on bactericidal activity was observed. Collectively, these data suggested that neutrophils from *Slc11a1*^*+/+*^ *Cebpe*^*−/−*^ mice were impaired in SLC11A1-mediated control of *Salmonella* infection. This SLC11A1-dependent effect of *Cebpe* deficiency on the control of intracellular *Salmonella* replication ex vivo was not observed in thioglycolate-elicited peritoneal macrophages (Fig. 4d), showing that C/EBPϵ deficiency does not affect the ability of these macrophages to control *Salmonella* infection. Using mice with different combinations of *Cebpe* and *Slc11a1* alleles, we then assessed the contribution of SLC11A1 in neutrophils to controlling systemic *S*. Typhimurium infection (Fig. 4e,f). In mice with functional SLC11A1, deficiency of C/EBPϵ compromised control of *Salmonella* in the spleen (Fig. 4e) and blood (Fig. 4f), while in the SLC11A1-deficient background, C/EBPϵ deficiency had a much reduced effect on systemic control of *Salmonella*. The finding that mice (that is, *Slc11a1*^*+/+*^ *Cebpe*^*−/−*^) exhibiting impaired *Slc11a1-*dependent host control in neutrophils (Fig. 4c) but not in macrophages (Fig. 4d) exhibited impaired control of systemic *Salmonella* infection (Fig. 4e,f) provided compelling support for the idea that neutrophils contribute to SLC11A1-mediated control of an intracellular bacterial pathogen.

## Discussion

Vitamin A has long been appreciated to play a role in immunity to infection^39^. Its active metabolite, retinoic acid, is essential for mucosal immunity in the intestine, including induction of adaptive immunity via recruitment of B and T cells^40–42^ and influencing the lineage development of mucosal T cells^42–45^ to modulate inflammatory responses. This study revealed a requirement of dietary vitamin A for expression of *Slc11a1* during terminal development of neutrophils. This is consistent with the known role of retinoic acid in the expression of *Cebpe*, which encodes a transcription factor required for terminal neutrophil differentiation^36^. Considering the broad role of retinoic acid in host physiology and transcriptional regulation, however, it is likely that additional effects of VAD on the immune system remain to be identified.

The function of SLC11A1 has been studied in macrophages, in which it mediates nutritional immunity to vacuolar pathogens via metal withholding, thereby limiting intracellular pathogen replication^33,46^. Here we show that an SLC11A1-dependent antimicrobial activity of neutrophils is required for controlling systemic *S*. Typhimurium infection. *S*. Typhimurium resides within macrophages during its growth in the liver and spleen, but some macrophages are killed, leading to extracellular release of the pathogen, making it susceptible to neutrophil attack^47^. The idea that an antimicrobial activity of neutrophils is necessary to eliminate extracellular *S*. Typhimurium is further supported by electron microscopic analysis of infected tissue in neutropenic mice^48^. Therefore, our results are consistent with the idea that SLC11A1 is important for both macrophage- and neutrophil-mediated control of *S*. Typhimurium infection. However, these findings raise the question whether the mechanism of SLC11A1-mediated host defence is the same in both macrophages and neutrophils. SLC11A1-dependent metal withholding would be expected to be bacteriostatic. SLC11A1 is found in the phagolysosomal membrane and mediates extrusion of divalent metals from the bacteria-containing phagosome of macrophages. While it has long been assumed that transport of iron and manganese out of the phagosome by SLC11A1 starves bacteria of these essential metal cofactors and prevents their intracellular replication, recent work suggests that SLC11A1 may actually limit bacterial access to a different metal cofactor, magnesium^29^. In neutrophils, SLC11A1 appears to be important in maximizing bactericidal activity, but it is unclear how its expression and activity promote bacterial killing by these cells, and future work is needed to address this question.

The finding that SLC11A1 supports neutrophil-mediated host defences has important implications for immunity to infection. Most mouse models of disease use C57BL/6 mice that carry the deficient *Slc11a1*^*D169*^ allele because of the broad availability of genetic tools for this background. However, while SLC11A1 has been linked genetically to multiple human autoimmune and infectious diseases, studies of its function have been limited primarily to restriction of intramacrophage pathogens. The role of SLC11A1 for neutrophil antibacterial function shown here suggests that it may be important in other infectious and inflammatory diseases in which neutrophil activity plays a pivotal role.

## Methods

### Mouse strains

All procedures were performed with 8–10-week-old C57BL/6 *Slc11a1*^*+/+*^, *Slc11a1*^*D169/D169*^, *Slc11a1*^*+/+*^ *Cepbe*^*+/+*^, *Slc11a1*^*+/+*^ *Cepbe*^*−/−*^, *Slc11a*^*D169/D169*^ *Cepbe*^*+/+*^ or *Slc11a*^*D169/D169*^ *Cepbe*^*−*^^*/*^^−^ mice. Male and female mice were used for each experiment unless otherwise specified in the figure legend. *Slc11a1*^*+/+*^ mice were obtained from G. Barton^22^, rederived into the barrier facility at UC Davis, backcrossed once with C57BL/6J mice and then bred and maintained under specific pathogen-free conditions by the UC Davis Teaching and Research Animal Care Service. Specific pathogen-free *Slc11a1*^*D169/D169*^ mice were purchased from The Jackson Laboratory (Bar Harbor, Maine). In addition, timed-pregnant female *Slc11a1*^*D169/D169*^ mice were purchased from The Jackson Laboratory and the pups were raised in the barrier facility at UC Davis to obtain VAD *Slc11a1*^*D169/D169*^ mice. *Cebpe* mice^37^ were provided by H. Phillip Koeffler and were backcrossed onto C57BL/6J and C57BL/6J-*Slc11a1*^*+/+*^ mice. All mice were held in microisolator cages with sterile ALPHA-dri bedding and received irradiated rodent feed and sterile drinking water ad libitum. The UC Davis Institutional Animal Care and Use Committee approved all animal experiments described in this paper under protocols 22492 and 23360.

### VAD mice and mice with sufficient vitamin A

Starting at 14 days of gestation, dams were fed a VAD diet (0 IU vitamin A kg^−1^). At weaning, mice were either placed on a diet replete with vitamin A (4,000 IU vitamin A kg^−1^) or maintained on the VAD diet. All diets were semi-purified and casein based. A custom VAD diet (TD.88407) and a vitamin A control diet with added orange food colouring (TD.09062) were prepared and pelleted by Envigo Teklad Diets. For vitamin A supplementation, VAD and control mice were treated with either 0.1 ml of sterile phosphate-buffered saline (PBS) or 600 IU retinyl palmitate (Nutrisorb A, Interplexus) in 0.1 ml of PBS by oral gavage at 7 and 3 days before infection with *S*. Typhimurium. For vitamin A treatment, VAD and control mice were treated with either 0.1 ml of sterile PBS or 600 IU retinyl palmitate in 0.1 ml of PBS by oral gavage starting 1 day after *S*. Typhimurium infection.

### Measurement of hepatic vitamin A levels

About 20 mg (control) to 50 mg (VAD) of the liver were homogenized with 100 mg sodium sulfate. The homogenate was transferred into a 7 ml glass vial and mixed with 350 µl ethanol, containing 0.1% butylated hydroxytoluol, for protein precipitation. After 100 µl of potassium hydroxide (KOH, 30% in deionized water) and 50 µl pyrogallol (10% in ethanol) were added, the samples were mixed for 15 s and incubated for 60 min at 60 °C to release the retinol from its retinyl esters. After the samples were cooled on ice, 3 ml hexane, 1 ml tocol (internal standard; 1 µg ml^−1^ in hexane) and 700 µl deionized water were added, mixed vigorously for 30 s and centrifuged for 2 min at 1,800 rpm for phase separation. The upper, organic phase was transferred into a fresh 7 ml glass vial and evaporated to dryness under a gentle nitrogen stream. The residue was reconstituted in 100 µl (VAD liver) or 200 µl (control liver) of acetonitrile before analysis. The analysis was carried out using an Agilent 1100 HPLC system equipped with a diode array detector (Agilent) controlled by OpenLABS ChemStation software (Rev A.01.04, Agilent). Samples were kept at 10 °C, and 10 µl was injected onto a Spherisorb ODS2 column, 125 × 3 mm, 3 µ (Waters) protected by a BDS-hypersil-C18 guard column, 20 × 3 mm, 3 µ (Thermo Scientific) at 15 °C. Acetonitrile, dichloromethane and methanol (7/2/1, v/v/v, all HPLC grade) served as isocratic mobile phase at a flow rate of 0.6 ml min^−1^ for 6 min. The detector was set at 325 nm for retinol and 295 nm for tocol, and quantification was carried out by ratio response to the internal standard.

### LPS challenge

Lipopolysaccharide from *E. coli* 0111:B4 strain (Invivogen catalogue code tlrl-eblps) was diluted in sterile, 0.9% sodium chloride, and 20 µg per mouse was administered intraperitoneally in a final volume of 100 µl. Male and female mice on control (*n* = 5) or VAD (*n* = 6) diets were killed 2 h after injection. Blood samples were collected in K_2_ ethylenediaminetetraacetic acid (EDTA) tubes, and plasma was collected and stored at −80 °C until further analysis. Cytokine levels were assessed with enzyme-linked immunosorbent assays for TNF-α (BioLegend catalogue number 430904) and IL-6 (Invitrogen catalogue number 88-7064).

### *S.* Typhimurium

A derivative of the *S*. Typhimurium clinical isolate D23580 Nal^R^ (*gyrA* S83F) pSLT-14028s::*tetRA*, designated JK1128, was provided by F. Fang and was used for animal infection studies^20^. Mice received either 0.1 ml of sterile PBS or 1 × 10^3^ colony-forming units (CFU) diluted in PBS by IP injection. For oral infection, mice received either 0.1 ml of Luria–Bertani (LB) broth or 1 × 10^9^ CFU diluted in LB broth by IG gavage. Inocula were cultured for 16–18 h aerobically at 37 °C. To determine tissue loads of viable *S*. Typhimurium, liver and spleen tissues were homogenized in PBS using an Ultra Turrax T25 basic mixer (IKA). Blood was collected by cardiac puncture with heparinized needles; plasma was removed and then incubated with 120 μl of 1% Triton X-100 in PBS for 10 min at room temperature. Homogenates were serially diluted and plated on LB agar plates containing 100 mg l^−1^ nalidixic acid (Sigma). After overnight growth at 37 °C, CFU g^−1^ or CFU ml^−1^ was calculated.

### RNA extraction, reverse-transcription PCR and real-time PCR

For whole-tissue RNA extractions, samples were snap-frozen in liquid nitrogen at time of necropsy and stored at −80 °C. RNA isolation from purified bone marrow neutrophils was performed on the same day. RNA was extracted from samples using Tri-Reagent (Molecular Research Center) according to the manufacturer’s instructions. All RNA samples were treated with DNase I (Ambion) to remove genomic DNA contamination. For quantification of messenger RNA (mRNA) levels in spleen tissue, 1 μg of total RNA from each sample was reverse transcribed in a 50 μl volume (TaqMan reverse transcription (RT) reagent; Applied Biosystems), and 4 μl of the resulting complementary DNA (cDNA) was used for each real-time reaction. For mRNA quantification from purified bone marrow neutrophils, 0.8 μg of total RNA from each sample was reverse transcribed in a 50 μl volume (TaqMan RT reagent; Applied Biosystems) and 4 μl of the resulting cDNA was used for each real-time reaction. Real-time PCR was performed using the primers listed in Table 1, SYBR green (Applied Biosystems) and ViiA 7 Real-Time PCR System (Applied Biosystems). Target gene transcription of each sample was normalized to the respective levels of β-actin (ACTB) or 18S rRNA, and absolute quantification was determined using gene-specific plasmid standards in each run.

**Table 1:** Quantitative real-time PCR primers used in this study

Target gene	Method	Sequences (5′–3′)
*Actb*	Absolute copies	CCAGGGAGGAAGAGGATGCGG
		GCTGAGAGGGAAATCGTGCGTG
*Cxcl1 (Kc)*	Absolute copies	GCTTGCCTTGACCCTGAAGCTC
		TGTTGTCAGAAGCCAGCGTTCAC
*Cxcl2 (Mip2)*	Absolute copies	CGCCCAGACAGAAGTCATAGCCAC
		TCCTTTCCAGGTCAGTTAGCCTTGC
18S rRNA	Absolute copies	GGCCGTTCTTAGTTGGTGGAGCG
		CTGAACGCCACTTGTCCCTC
*Mpo*	Absolute copies	GGAAGGAGACCTAGAGGTTGG
		TAGCACAGGAAGGCCAATG
*Mmp9*	Absolute copies	ACGACATAGACGGCATCCA
		TGTCGGCTGTGGTTCAGTT
*Ltf*	Absolute copies	TGCTTGCTAACCAGACCAGA
		ACCAATACACAGGGCACAGA
*Csf3r*	Absolute copies	CTGATCTTCTTGCTACTCCCCA
		GGTGTAGTTCAAGTGAGGCAG
*Slc11a1*	Absolute copies	TACCAGCAAACCAATGAGGA
		CCTGGGGAAGATCTTAGCATAGT

### Western blot

Protein was extracted from bone marrow neutrophils of control and VAD mice using Tri-Reagent (Molecular Research Center) according to the manufacturer’s instructions. The concentration of bone marrow neutrophil protein was measured using a modified Bradford assay. Briefly, samples were diluted in 0.15 M NaCl and 1 ml of Bradford substrate (0.1 mg ml^−1^ Coomassie Brilliant Blue G-250, 5% ethanol and 10% of 85% (w/v) phosphoric acid) was added to 100 μl of either sample of standard. A 10 μg sample of protein was separated using sodium dodecyl sulfate-polyacrylamide gel electrophoresis and transferred to a polyvinylidene fluoride membrane (Millipore). A blocking solution of 2.5% non-fat dried milk and 0.1% Tween 20 (Bio-Rad) in PBS was used. For lactoferrin detection, a 1:200 dilution of the primary antibody (lactoferrin (H-65) rabbit polyclonal IgG, catalogue number sc-25622, Santa Cruz Biotechnology) in blocking solution was added to the membrane. As a loading control, glyceraldehyde 3-phosphate dehydrogenase (GAPDH) was detected at a 1:5,000 dilution of the primary antibody (GAPDH rabbit mAb, clone 14C10, catalogue number 2118S, Cell Signaling) in blocking solution. A goat anti-rabbit horseradish peroxidase conjugated secondary antibody (Bio-Rad) was diluted 1:3,000 in blocking buffer and applied to the membrane. All antibodies were validated by the manufacturers. Protein bands were visualized by chemiluminescence (SuperSignal West Femto Maximum Sensitivity Substrate, ThermoFisher Scientific) using a BioSpectrum (UVP) imaging system. Raw images were processed using Photoshop CS2 (Adobe Systems) to uniformly adjust brightness.

### Isolation of neutrophils from mouse bone marrow

Bone marrow fractionation was performed using a modification of the density gradient centrifugation method previously described^49^. Briefly, bone marrow was flushed from the femora and tibiae with 10 ml of sterile PBS and passed through an 18-gauge needle to disrupt larger bone marrow clumps. Cells were centrifuged at 300 × *g* for 7 min at 4 °C. Red blood cells were lysed by resuspending a cell pellet in 0.2% NaCl for 20 s followed by the addition of 1.6% NaCl. Cells were centrifuged at 300 × *g* for 7 min at 4 °C, washed with 2 mM EDTA in PBS and filtered through a 40 μm filter. Using a 15 ml conical tube, 3 ml of Histopaque 1119 (density 1.119 g ml^−1^, Sigma-Aldrich) was overlaid with 3 ml of Histopaque 1077 (density 1.077 g ml^−1^, Sigma-Aldrich). Bone marrow cells were resuspended in 1 ml of ice-cold PBS and laid over the Histopaque gradient. Samples were centrifuged for 30 min at 700 × *g* at 25 °C without a break. Neutrophils were collected at the interface of the Histopaque 1119 and Histopaque 1077 layers and then washed twice with PBS and used for further experiments. The composition of the cell population was confirmed using microscopy to have neutrophil morphology as determined by Giemsa staining.

### Spleen neutrophil enrichment

Neutrophils were enriched from the spleen of mice infected with *S*. Typhimurium using the EasySep Mouse Neutrophil Enrichment Kit (STEMCELL Technologies) according to the manufacturer’s protocol. Briefly, spleens were removed aseptically and smashed using a syringe plunger to produce a single-cell suspension. Red blood cells were lysed by the addition of ACK lysing buffer (Lonza). Cells were centrifuged at 600 × *g* for 10 min at 4 °C, washed twice with Dulbecco’s PBS (dPBS) and filtered using a 70 mm filter. Neutrophils were enriched by immunomagnetic negative selection. For *S*. Typhimurium counts, purified neutrophils were enumerated from each sample and neutrophils were lysed with 1% Triton X-100 in PBS or radioimmunoprecipitation assay buffer for 10 min at room temperature. The suspension was serially diluted and plated on LB agar plates containing 100 mg l^−1^ nalidixic acid (Sigma-Aldrich). After overnight growth at 37 °C, bacterial counts were calculated as CFU per 10^5^ or CFU per 5 × 10^5^ neutrophils.

### Flow cytometry

Flow cytometry analysis was performed for the detection of neutrophils in the spleen and bone marrow of control and VAD mice mock infected and infected with *S*. Typhimurium. Single-cell suspensions of spleen and bone marrow tissue were obtained as described previously. Cells were resuspended in 2 ml of dPBS and stained with an Aqua Live/Dead cell discriminator (Invitrogen) according to the manufacturer’s protocol. After Live/Dead staining, cells were washed with dPBS and resuspended in 50 μl of PBS containing 1% bovine serum albumin and 2 mM EDTA (fluorescence-activated cell sorter (FACS) buffer). Cells were stained with an Fc receptor blocking antibody, anti-CD16/32 (93) (eBioscience), for 5 min at 4°C and then stained for 20 min at 4°C with a cocktail of anti-B220 (RA3-6B2) PerCp-Cy5.5, anti-CD19 (6D5) PerCp-Cy5.5, anti-CD8a (53–6.7) PerCyp-Cy5.5, anti-CD4 (RM4-5) PerCp-Cy5.5, anti-CD11b (M1/70) PE-Cy7, anti-Ly6G (1A8) APC and anti-Ly6C (HK1.4) Pacific Blue (all BioLegend). In addition, for bone marrow samples, an anti-Ter119 (TER-119) PerCp-Cy5.5 (BioLegend) was added. All antibodies were validated by the manufacturers. Cells were washed twice in FACS buffer, fixed with BD Cytofix (BD Biosciences) for 30 min at 4 °C and resuspended in FACS buffer. For quantification of cell populations, 50 μl of SPHERO AccuCount Fluorescent Particles 10.1 μm (Spherotech) was added to each sample before analysis. Calculation of absolute counts was performed according to the manufacturer’s protocol. Flow cytometry analysis was performed using a BD (Becton Dickinson) LSRII, and 1.0 × 10^6^ events were collected per mouse. Data were analysed using FlowJo software (BD Biosciences), and gates were based on fluorescence-minus-one controls.

### ELISA

The levels of IL-6 and TNF-α in serum samples from control and VAD mice were determined by enzyme-linked immunosorbent assay (ELISA) (R&D Systems), according to the manufacturer’s instructions. The ELISA test was read at 450 nm with an ELISA microplate reader (Bio-Rad Model 680). Data points are the averages of duplicate dilutions.

### In vivo depletion of neutrophils

For neutrophil depletion experiments, control and VAD male mice were injected intraperitoneally 1 day before and after S. Typhimurium infection with either 500 μg of rat anti-mouse Ly6G monoclonal antibody, clone 1A8 (BioXCell), or 500 μg of rat IgG2a isotype control, clone 2A3 (BioXCell), diluted in 0.2 ml of dPBS. Neutrophil depletion in the tissue was confirmed by flow cytometry as described previously.

### Adoptive transfer

Bone marrow neutrophils were isolated from male and female 8–12-week-old, healthy C57BL/*6 Slc11a1*^*+/+*^ mice using the protocol described above. Once collected, neutrophils were washed twice with dPBS, counted and suspended in dPBS at a concentration of 1.5–2 × 10^7^ cells ml^−1^. Male VAD mice were inoculated intragastrically 1 day after *S*. Typhimurium infection with either 0.2 ml of the neutrophil suspension (total of 3–4 × 10^6^ neutrophils per mouse) or 0.2 ml of dPBS. Animals were necropsied 3 days after *S*. Typhimurium infection to assess bacteriology.

### Statistical analysis

The statistical significance of differences between groups was determined by a one- or two-tailed Student’s *t*-test, or one-way analysis of variance (ANOVA) with a post hoc Tukey or Sidak’s test on logarithmically or arc-sin-transformed data. A *P* value of 0.05 or less was considered to be significant. Animals were excluded if they were not confirmed to be infected after intraperitoneal (IP) administration of *S*. Typhimurium. Data points that were identified as outliers were excluded based on the ROUT method. No statistical methods were used to predetermine sample sizes, but our sample sizes are similar to those reported in previous publications^23^. GraphPad Prism 6 was used to perform analyses (GraphPad).

### Reporting summary

Further information on research design is available in the Nature Portfolio Reporting Summary linked to this article.

### Supplementary information

Supplementary Information Extended Data Fig. 2b uncropped western blots.

Reporting Summary

### Source data

Source Data Fig. 1 Source data for all figure panels.

Source Data Fig. 2 Source data for all figure panels.

Source Data Fig. 3 Source data for all figure panels.

Source Data Fig. 4 Source data for all figure panels.

Source Data Extended Data Fig. 1 Source data for all figure panels.

Source Data Extended Data Fig. 2 Source data for all figure panels.

Source Data Extended Data Fig. 3 Source data for all figure panels.

Source Data Extended Data Fig. 4 Source data for all figure panels.

## Data Availability

All data supporting the findings of this study are available within the article and its Supplementary Information. Additional data supporting the findings in this study are available from the corresponding author upon request. Source data are provided with this paper.

## Code availability

No code was used to analyse any of the data in this paper.
