# Supplementary material for: Vitamin A deficiency impairs neutrophil-mediated control of Salmonella via SLC11A1 in mice
Source: Nat Microbiol. 2024 Feb 19;9(3):727–36. doi: 10.1038/s41564-024-01613-0 (PMC10914596; doi:10.1038/s41564-024-01613-0)

nature microbiology

Article https://doi.org/10.1038/s41564-024-01613-0

Vitamin A deficiency impairs neutrophil-mediated control of *Salmonella* via SLC11A1 in mice

In the format provided by the authors and unedited

Source data for Extended Data Fig 2b

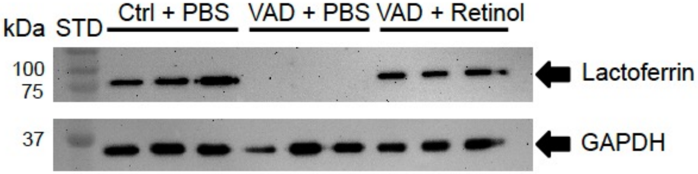

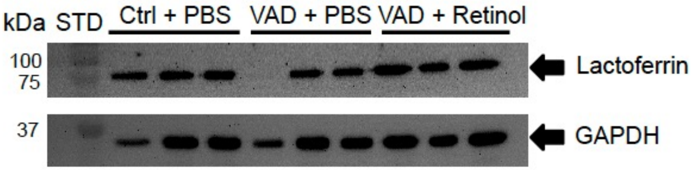

Overlay of adjusted blots | Adjusted blots | Overlay of adjusted blots | Adjusted blots

Lactoferrin

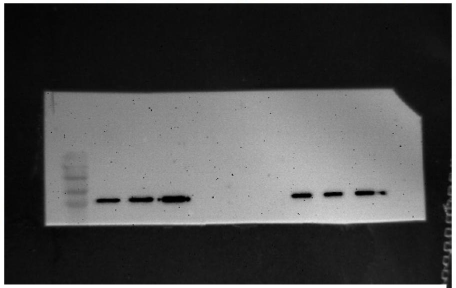

Resolved protein standards bottom to top (kDa): 75, 100, 150

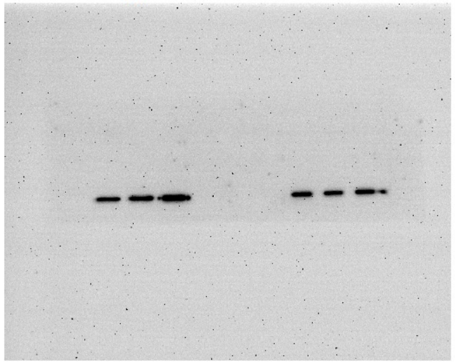

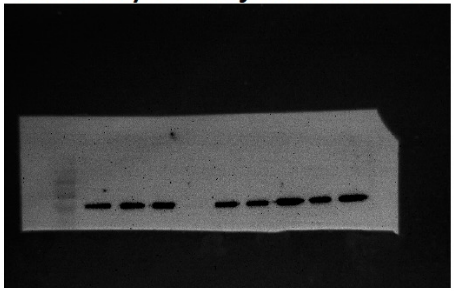

Resolved protein standards bottom to top (kDa): 75, 100, 150

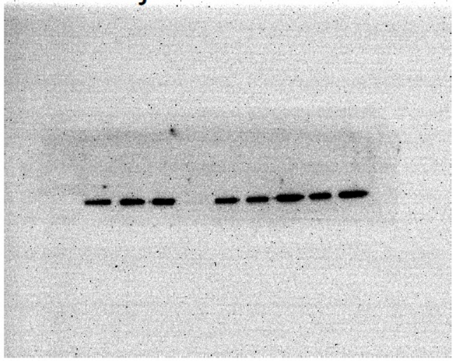

GAPDH

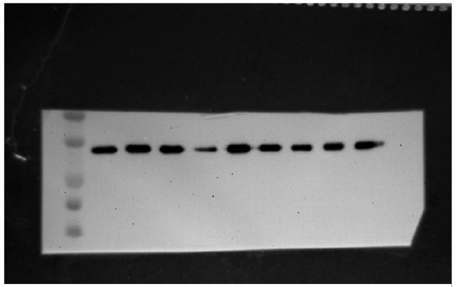

Protein standards bottom to top (kDa): 15, 20, 25, 37, 50

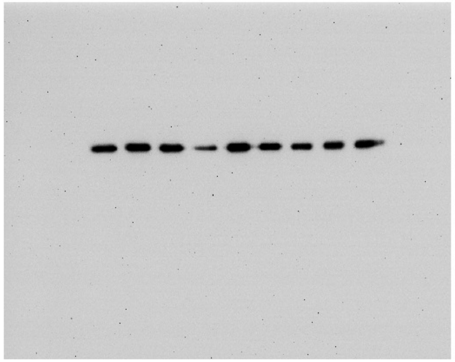

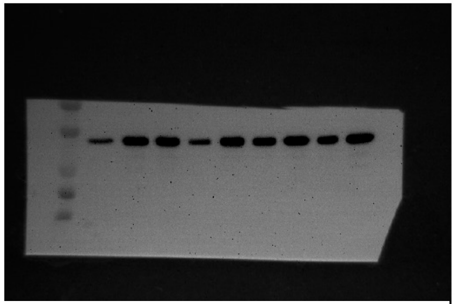

Protein standards bottom to top (kDa): 15, 20, 25, 37, 50

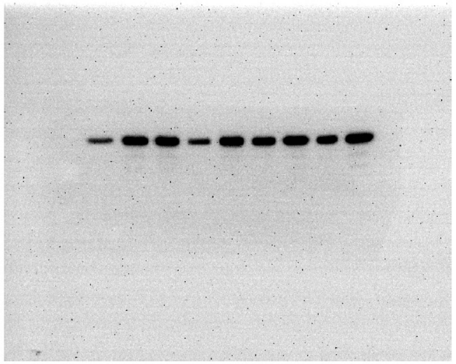

Supplement: Supplementary file 1 — Extended Data Fig. 2b uncropped western blots. [file 41564_2024_1613_MOESM1_ESM.pdf]
