# Supplementary material for: Vitamin A deficiency impairs neutrophil-mediated control of Salmonella via SLC11A1 in mice
Source: Nat Microbiol. 2024 Feb 19;9(3):727–36. doi: 10.1038/s41564-024-01613-0 (PMC10914596; doi:10.1038/s41564-024-01613-0)
Supplement: Supplementary file 2 — Reporting Summary [file 41564_2024_1613_MOESM2_ESM.pdf]

nature portfolio

Corresponding author(s): Tsolis, Renee M.

Last updated by author(s): Jan 11, 2024

Reporting Summary

Nature Portfolio wishes to improve the reproducibility of the work that we publish. This form provides structure for consistency and transparency in reporting. For further information on Nature Portfolio policies, see our Editorial Policies and the Editorial Policy Checklist.

Statistics

For all statistical analyses, confirm that the following items are present in the figure legend, table legend, main text, or Methods section.

n/a	Confirmed	
☐	☒	The exact sample size (*n*) for each experimental group/condition, given as a discrete number and unit of measurement
☐	☒	A statement on whether measurements were taken from distinct samples or whether the same sample was measured repeatedly
☐	☒	The statistical test(s) used AND whether they are one- or two-sided<br>*Only common tests should be described solely by name; describe more complex techniques in the Methods section.*
☒	☐	A description of all covariates tested
☒	☐	A description of any assumptions or corrections, such as tests of normality and adjustment for multiple comparisons
☐	☒	A full description of the statistical parameters including central tendency (e.g. means) or other basic estimates (e.g. regression coefficient) AND variation (e.g. standard deviation) or associated estimates of uncertainty (e.g. confidence intervals)
☐	☒	For null hypothesis testing, the test statistic (e.g. *F*, *t*, *r*) with confidence intervals, effect sizes, degrees of freedom and *P* value noted<br>*Give P values as exact values whenever suitable.*
☒	☐	For Bayesian analysis, information on the choice of priors and Markov chain Monte Carlo settings
☒	☐	For hierarchical and complex designs, identification of the appropriate level for tests and full reporting of outcomes
☒	☐	Estimates of effect sizes (e.g. Cohen's *d*, Pearson's *r*), indicating how they were calculated

Our web collection on statistics for biologists contains articles on many of the points above.

Software and code

Policy information about availability of computer code

Data collection	No original software or code was used for data collection
Data analysis	Graph Pad Prism 10, FlowJo

For manuscripts utilizing custom algorithms or software that are central to the research but not yet described in published literature, software must be made available to editors and reviewers. We strongly encourage code deposition in a community repository (e.g. GitHub). See the Nature Portfolio guidelines for submitting code & software for further information.

Data

Policy information about availability of data

All manuscripts must include a data availability statement. This statement should provide the following information, where applicable:

- Accession codes, unique identifiers, or web links for publicly available datasets
- A description of any restrictions on data availability
- For clinical datasets or third party data, please ensure that the statement adheres to our policy

All data supporting the findings of this study are available within the Article, and its Supplementary Information. Additional data supporting the findings in this study are available from the corresponding authors upon request.

Research involving human participants, their data, or biological material

Policy information about studies with human participants or human data. See also policy information about sex, gender (identity/presentation), and sexual orientation and race, ethnicity and racism.

Reporting on sex and gender	N/A
Reporting on race, ethnicity, or other socially relevant groupings	N/A
Population characteristics	N/A
Recruitment	N/A
Ethics oversight	N/A

Note that full information on the approval of the study protocol must also be provided in the manuscript.

Field-specific reporting

Please select the one below that is the best fit for your research. If you are not sure, read the appropriate sections before making your selection.

☒ Life sciences ☐ Behavioural & social sciences ☐ Ecological, evolutionary & environmental sciences

For a reference copy of the document with all sections, see nature.com/documents/nr-reporting-summary-flat.pdf

Life sciences study design

All studies must disclose on these points even when the disclosure is negative.

Sample size	*Describe how sample size was determined, detailing any statistical methods used to predetermine sample size OR if no sample-size calculation was performed, describe how sample sizes were chosen and provide a rationale for why these sample sizes are sufficient.*
Data exclusions	Animals were excluded from our analysis if they were not confirmed to be infected after IP administration of S. Typhimurium. Data points that were identified as outliers were excluded based on the ROUT method.
Replication	In vitro experiments were replicated three times independently with triplicate samples. In vivo experiments were performed once per experimental design and the results were replicated with a related experimental design.
Randomization	Mice were allocated randomly to experimental groups.
Blinding	Blinding was not relevant to our study, as the data generated were solely quantitative in nature.

Reporting for specific materials, systems and methods

We require information from authors about some types of materials, experimental systems and methods used in many studies. Here, indicate whether each material, system or method listed is relevant to your study. If you are not sure if a list item applies to your research, read the appropriate section before selecting a response.

Materials & experimental systems

n/a	Involved in the study	
☐	☒	Antibodies
☒	☐	Eukaryotic cell lines
☒	☐	Palaeontology and archaeology
☐	☒	Animals and other organisms
☒	☐	Clinical data
☒	☐	Dual use research of concern
☒	☐	Plants

Methods

n/a	Involved in the study	
☒	☐	ChIP-seq
☐	☒	Flow cytometry
☒	☐	MRI-based neuroimaging

Antibodies

Antibodies used	anti-CD16/32, anti-B220 (RA3-6B2) PerCp-Cy5.5,anti-CD19 (6D5) PerCp-Cy5.5,anti-CD8a (53-6.7) PerCyp-Cy5.5, anti-CD4 (RM4-5)

PerCp-Cy5.5, anti-CD11b (M1/70) PE-Cy7, anti-Ly6G (1A8) APC, and anti-Ly6C (HK1.4) Pacific Blue (all BioLegend, San Diego, CA, USA)
anti-Ter119 (TER-119) PerCp-Cy5.5 (BioLegend)
rat anti-mouse Ly6G monoclonal antibody, clone 1A8 (BioXCell, West Lebanon, NH, USA)
GAPDH rabbit mAb, clone 14C10, cat. Number 2118S, Cell Signaling, used at 1:5000
goat anti-rabbit horseradish peroxidase (HRP) conjugated secondary antibody (Bio-Rad) used at 1:3000
lactoferrin (H-65) rabbit polyclonal mAb, cat. number sc-25622, Santa Cruz Biotechnology INC., Santa Cruz, CA, used at 1:200
rat IgG2a isotype control, clone 2A3 (BioXCell)

Validation

Validation statement for GAPDH rabbit mAb https://www.cellsignal.com/products/primary-antibodies/gapdh-14c10-rabbit-mab/2118
Lactoferrin (H-65) has been discontinued by the manufacturer and replaced by another product. The validation statement is no longer available.

Animals and other research organisms

Policy information about studies involving animals; ARRIVE guidelines recommended for reporting animal research, and Sex and Gender in Research

Laboratory animals

All procedures were performed with 8-10 week-old C57BL/6 Slc11a1+/+, Slc11a1D169/D169, Slc11a1+/+ Cepbe+/+, Slc11a1+/+ Cepbe-/-, Slc11aD169/D169 Cepbe+/+ or Slc11aD169/D169 Cepbe-/- mice. Male and female mice were used for each experiment unless otherwise specified in the figure legend.

Wild animals

No wild animals were used in this study

Reporting on sex

Sex of all animals used is reported in the legend for each figure. When sex-specific effects of treatments were observed after disaggregating data by sex, data from both male and female mice are presented separately.

Field-collected samples

No field-collected samples were used.

Ethics oversight

Studies with mice were approved by the UC Davis Institutional Animal Care and Use Committee.

Note that full information on the approval of the study protocol must also be provided in the manuscript.

Plants

Seed stocks

Not applicable

Novel plant genotypes

Not applicable

Authentication

Not applicable

Flow Cytometry

Plots

Confirm that:

☒ The axis labels state the marker and fluorochrome used (e.g. CD4-FITC).

☒ The axis scales are clearly visible. Include numbers along axes only for bottom left plot of group (a 'group' is an analysis of identical markers).

☒ All plots are contour plots with outliers or pseudocolor plots.

☒ A numerical value for number of cells or percentage (with statistics) is provided.

Methodology

Sample preparation

Bone marrow: Bone marrow was flushed from the femora and tibiae with 10 ml of sterile PBS and passed through an 18-gauge needle to disrupt larger bone marrow clumps. Cells were centrifuged at 300 x g for 7 min at 4C. Red blood cells were lysed by resuspending cell pellet in 0.2% NaCl for 20 seconds followed by addition of 1.6% NaCl. Cells were centrifuged at 300 x g for 7 min at 4C, washed with 2 mM EDTA in PBS and filtered through a 40 m filter. Using a 15 ml conical tube, 3 ml Histopaque 1119 (density 1.119 g/ml, Sigma-Aldrich, St. Louis, MO, USA) was overlaid with 3 ml of Histopaque 1077 (density 1.077 g/ml, Sigma-Aldrich). Bone marrow cells were resuspended in 1 ml of ice-cold PBS and laid over the Histopaque gradient. Samples were centrifuged for 30 min at 700 x g at 25C without break. Neutrophils were collected at the interface of Histopaque 1119 and Histopaque 1077 layers and then washed twice with PBS and used for further experiments. The composition of the cell population was confirmed by microscopy to have neutrophil morphology as determined by Giemsa

staining.

Spleen:
bone marrow was flushed from the femora and tibiae with 10 ml of sterile PBS and passed through an 18-gauge needle to disrupt larger bone marrow clumps. Cells were centrifuged at 300 x g for 7 min at 4C. Red blood cells were lysed by resuspending cell pellet in 0.2% NaCl for 20 seconds followed by addition of 1.6% NaCl. Cells were centrifuged at 300 x g for 7 min at 4C, washed with 2 mM EDTA in PBS and filtered through a 40 m filter. Using a 15 ml conical tube, 3 ml Histopaque 1119 (density 1.119 g/ml, Sigma-Aldrich, St. Louis, MO, USA) was overlaid with 3 ml of Histopaque 1077 (density 1.077 g/ml, Sigma-Aldrich). Bone marrow cells were resuspended in 1 ml of ice-cold PBS and laid over the Histopaque gradient. Samples were centrifuged for 30 min at 700 x g at 25C without break. Neutrophils were collected at the interface of Histopaque 1119 and Histopaque 1077 layers and then washed twice with PBS and used for further experiments. The composition of the cell population was confirmed by microscopy to have neutrophil morphology as determined by Giemsa staining.

Instrument

Flow cytometry analysis was performed using a BD (Becton Dickinson, East Rutherford, NJ, USA) LSRII

Software

Data were analyzed using FlowJo software (Treestar, Inc. Ashland, OR) and gates were based on fluorescence-minus-one (FMO) controls.

Cell population abundance

We did not analyze post-sort fractions, as cell sorting was not performed.

Gating strategy

After doublet elimination, live cells were gated and CD4-CD8a-CD19-B220- Ter119-, for bone marrow analysis, and CD4-CD8a-CD19-B220- for spleen analysis, were analyzed for CD11b and Ly6G expression. Neutrophils were defined as CD11b +Ly6G+.

☒ Tick this box to confirm that a figure exemplifying the gating strategy is provided in the Supplementary Information.
